# Identification of key genes and functions of circulating tumor cells in multiple cancers through bioinformatic analysis

**DOI:** 10.1186/s12920-020-00795-w

**Published:** 2020-09-24

**Authors:** Yibing Guan, Fangshi Xu, Yiyuan Wang, Juanhua Tian, Ziyan Wan, Zhenlong Wang, Tie Chong

**Affiliations:** 1 Department of Urology, the Second Affiliated Hospital, School of Medicine, Xi’an Jiaotong University, No 157 Xiwu Road, Xi’an, 710004 Shaan Xi Province China; 2 School of Medicine, Xi’an Jiaotong University, Xi’an, China; 3 Department of Stomatology, the Second Affiliated Hospital of Shaanxi University of Traditional Chinese Medicine, Xianyang, China

**Keywords:** Hub genes, Expression profiling data, Cancer, PSMC2, EMT

## Abstract

**Background:**

Circulating tumor cells (CTCs) play a key role in cancer progression, especially metastasis, due to the rarity and heterogeneity of CTCs, fewer researches have been conducted on them at the molecular level. However, through the Gene Expression Omnibus (GEO) database, this kind of minority researches can be well integrated, the gene expression differences between CTCs and primary tumors can be identified, and molecular targets for CTCs can be found.

**Methods:**

We analyzed 7 sets of gene chips (GSE82198, GSE99394, GSE31023, GSE65505, GSE67982, GSE76250, GSE50746) obtained by GEO. Analysis of differentially expressed genes (DEGs) between CTCs and corresponding primary tumors by NetworkAnalyst. Metascape tool for Gene Ontology (GO) / Kyoto Encyclopedia of Genes and Genomes (KEGG) enrichment analysis of differential genes and visual display. Cytoscape performs protein-protein interaction (PPI) analysis and obtains the hub genes. Renal cancer patients’ clinical specimens to verify the correctness of enrichment results. Prognostic analysis of hub genes in kidney cancer patients using the Kaplan–Meier plotter survival analysis tool.

**Results:**

We obtained a total of 589 DEGs. The GO / KEGG enrichment results indicate that the DEGs are mainly concentrated in cell adhesion, epithelial-mesenchymal transition (EMT), and apoptosis. Renal cancer clinical specimens suggest that CTCs have epithelial and mesenchymal types. At the same time, PSMC2 can be used as a poor prognostic indicator for renal cancer patients.

**Conclusions:**

In summary, our study suggests that compared with primary tumors, CTCs mainly change cell adhesion, EMT, and apoptosis. PSMC2 can be used as a poor prognostic factor.

## Background

Circulating tumor cells, as cells detached from solid tumors and entering the circulating blood are a new type of tumor biomarker with real-time detection and minimally invasive characteristics [1]. At the same time, CTCs play an important role in tumor progression, especially in the formation of metastases [2]. CTCs have the characteristics of rarity and heterogeneity, which once restricted the researches of CTCs [3]. Nowadays, with the development of technology, not only can CTCs be captured and enriched, but also they can be identified at the molecular level, which greatly improves the clinical application value of CTCs, and provides potential possible for the prognosis of tumor patients and personalized treatment [4]. In this article, we analyze the differentially expressed genes (DEGs) between CTCs and primary tumors through the Gene Expression Omnibus (GEO) database and use Gene Ontology (GO) / Kyoto Encyclopedia of Genes and Genomes (KEGG) enrichment tools, protein-protein interaction (PPI) analysis tools, and Kaplan–Meier plotter tools to analyze cell biological process, cellular component, molecular function, biological pathways, protein interactions, and clinical prognosis. Identify the differences between CTCs and primary tumors, and provide potential targets for tumor prognosis and treatment.

## Methods

### Data sources

In this study, gene expression data were obtained from the GEO database (https://www.ncbi.nlm.nih.gov/geo/). After careful screening, a total of GSE82198, GSE99394, and GSE31023 were included in the analysis, GSE65505, GSE67982, GSE76250, GSE50746 were also used as primary tumor controls. GSE31023 and GSE50746 are based on the Agilent-026652 Whole Human Genome Microarray 4x44K v2 (Probe Name version) platform. GSE82198 is based on the [HG-U133_Plus_2] Affymetrix Human Genome U133 Plus 2.0 Array platform. GSE99394, GSE65505, GSE67982, GSE76250 are based on the [HTA-2_0] Affymetrix Human Transcriptome Array 2.0 [transcript (gene) version] platform. Free access to all data.

### Acquisition of DEGs

The NetworkAnalyst tool (https://www.networkanalyst.ca/) was used to analyze the expression of CTCs and corresponding primary tumor genes in the above data set, and then genes with adjusted *P* < 0.05 and | logFC | > 1 were marked as DEGs [5]. The NetworkAnalyst tool includes steps such as data filtering, normalization, and difference analysis. Through the above steps and the removal of the batch effect function of the limma package in the difference analysis, it can reduce the influence of batch effect on experimental results. After obtaining the DEGs of the three tumors, the DGEs co-expressed in different cancers were obtained through the Wayne Diagram tool (http://bioinformatics.psb.ugent.be/webtools/Venn/).

### GO / KEGG pathway analysis of DEGs

The metascape tool (https://metascape.org/gp/index.html#/main/step1) was used to perform GO biological processes (BP), cell components (CC), molecular functions (MF), and KEGG pathway enrichment analysis of 589 DEGs [6]. Screening for enrichment information with statistical significance at *P* < 0.05.

### PPI network construction and hub gene identification

The STRING tool (https://string-db.org/cgi/input.pl) was used to analyze the obtained 589 DEGs, set the confidence greater than 0.7 as the screening condition, and obtain the PPI network [7]. Use the Cytoscape tool (https://cytoscape.org/) to visualize the PPI network, and use the Cytohubba plugin CytoHubba to calculate the protein score and obtain the top 10 hub genes with the highest score [8].

### Patient CTCs analysis and hub gene survival analysis

With the approval of the ethics review committee of the Second Affiliated Hospital of Xi’an Jiaotong University, we obtained CTCs test data of renal cancer patients who had undergone surgical treatment in our hospital from September 2015 to January 2019. The studies using clinical samples were approved by the institutional review boards of the Second Affiliated Hospital of Xi’an Jiaotong University, and written-informed consents were obtained from all participants. Peripheral blood samples (5 ml, anticoagulated with EDTA) were drawn from all participants before each time point. Mononuclear cells (MNC) were isolated by adding erythrocyte lysis buffer (154 mM NH4Cl, 10 mM KHCO3 and 0.1 mM EDTA, Sigma, St. Louis, USA). After centrifugation (1500 rpm, 5 min), the blood samples were resuspended in PBS buffer. CTCs were separated by using CanPatrol CTC enrichment technique (SurExam, Guangzhou, China). CTCs were divided into epithelial, mesenchymal and mixed phenotype based on the morphological and biological biomarkers. Epithelial CTCs were detected with labeling epithelia markers, such as EpCAM, CK8, CK18 and CK19. Mesenchymal CTCs were tested by labeling mesenchymal markers, including Vimentin and Twist. The expression levels of EpCAM, CK8, CK18, CK19, Vimentin and Twist were detected by RNA in situ hybridization technique. The detailed procedures were described as following. White blood cells were loaded into 24 wells cell culture plates. All cells were treated with proteinase. Subsequently, targeted gene genomic DNA sequences were detected with capture probes including preamplifier sequence, the amplifier sequence and the labeled probe17. Epithelial markers EpCAM, CK8, CK18 and CK19 were labeled with Alexa Fluor 594. Mesenchymal markers Vimentin and Twist were labeled with Alexa Fluor 488. White blood cells were stained by Alexa Fluor 750 conjugated anti-CD45. Meanwhile, the Kaplan–Meier plotter tool was used to analyze the OS and RFS prognosis of hub genes in renal clear cell carcinoma. *P* < 0.05 was considered statistically significant.

### Expression of PSMC2 in kidney cancer cell lines and tissues

Access the RNAseq data of *PSMC2* in common kidney cancer cell lines through the Cancer Cell Line Encyclopedia (CCLE) database (https://portals.broadinstitute.org/ccle/about) [9]. Statistics of expression data through Prism software. Immunohistochemical pictures of *PSMC2* in normal kidney tissues and kidney cancer tissues were obtained from the human protein atlas (HPA) database (https://www.proteinatlas.org/) [10]. Both tissues were treated with the same antibody Antibody HPA019238. Quantitative analysis of immunohistochemical results through imageJ.

## Results

### Identification of DEGs

**Fig. 1:**
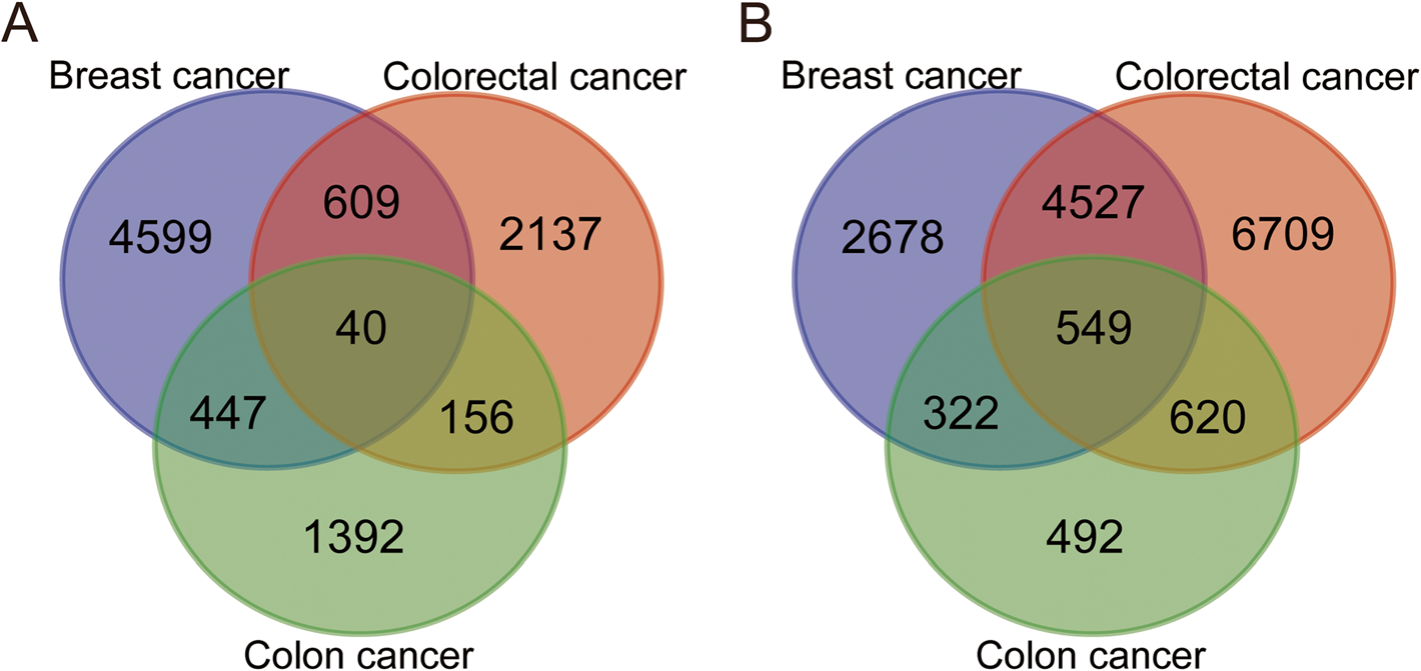
Venn diagram of three cancers with common DEGs. **a** Upregulated genes. **b** Downregulated genes

In this study, a total of 3 cancer species and 7 groups of gene expression were included (Table 1), of which 3 were CTCs samples of colon cancer, 3 were primary tumor samples (GSE82198), 10 were CTCs samples from breast cancer, and 9 were primary samples tumor samples (GSE99394, GSE65505, GSE67982, GSE76250), 6 CTCs samples from colorectal cancer, 7 primary tumor samples (GSE31023, GSE50746). Detailed sample information is shown in Table S3. With adjusted *P* < 0.05 and | logFC | > 1 as the screening conditions, a total of 5695 genes were up-regulated and 8076 genes were down-regulated in breast cancer. A total of 2942 genes were up-regulated and 12,405 genes were down-regulated in colorectal cancer. In colon cancer, a total of 2035 genes were up-regulated and 1983 genes were down-regulated. These DEGs were obtained by comparing the CTCs with the primary tumor. Subsequently, the links between the differential genes in the three cancers were analyzed using a Wayne diagram (Fig. 1). In the end, a total of 589 genes were differentially expressed in all three cancers, of which 40 were up-regulated and 549 were down-regulated.

**Table 1:** Statistics of the seven microarray databases derived from the GEO database

Cancer	Dataset ID	CTCs	Primary tumor	Total number
Colon cancer	GSE82198	3	3	6
Breast cancer	GSE99394	10	0	19
	GSE65505	0	3	
	GSE67982	0	3	
	GSE76250	0	3	
Colorectal cancer	GSE31023	6	0	13
	GSE50746	0	7	

**Abbreviations:** *GEO* Gene Expression Omnibus, *CTCs* Circulating tumor cells

### Functional enrichment analyses of DEGs

**Fig. 2:**
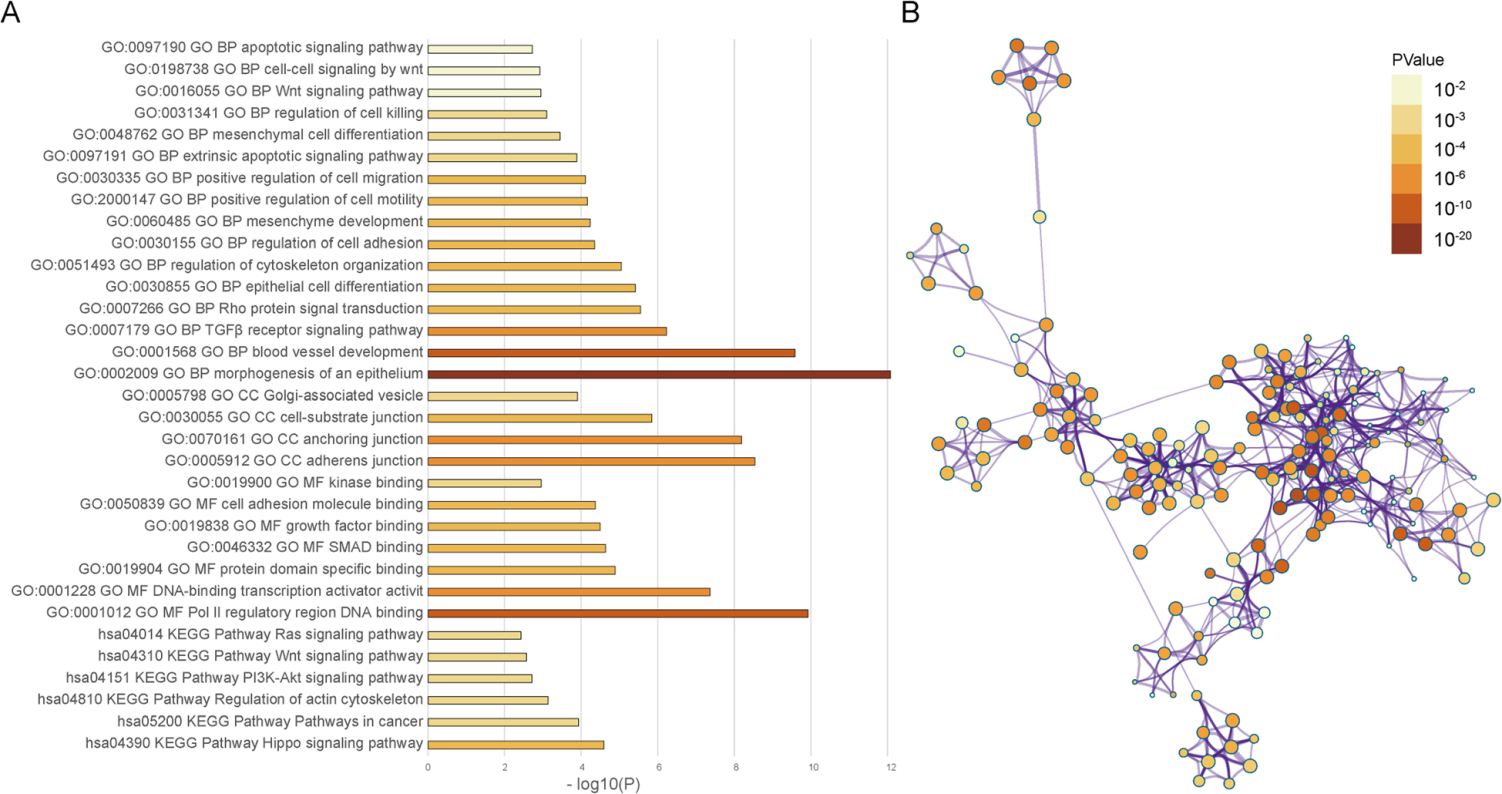
DEGs enrichment results. **a** Significantly enriched GO terms and KEGG pathways of DEGs. **b** Network contact of GO terms and KEGG pathways

The GO and KEGG pathway enrichment analysis of differential genes was performed by Metascape (Fig. 2). The enriched results are divided into 4 parts, BP, CC, MF, and KEGG analysis. In BP, DEGs are mainly concentrated in epithelial and mesenchymal differentiation, cytoskeleton, invasiveness, and changes in apoptosis. In CC, DEGs are mainly concentrated in the links between cells, and Golgi-related vesicles. In MF, DEGs are mainly concentrated in the activation of the binding of transcription factors to DNA, the binding of protein domains, the binding of growth factors, adhesion molecules, and kinases, etc. In the KEGG pathway, DEGs are mainly concentrated in signaling pathways such as Hippo, cancer, PI3K-Akt, and Wnt.

### PPI network construction and hub gene identification

**Fig. 3:**
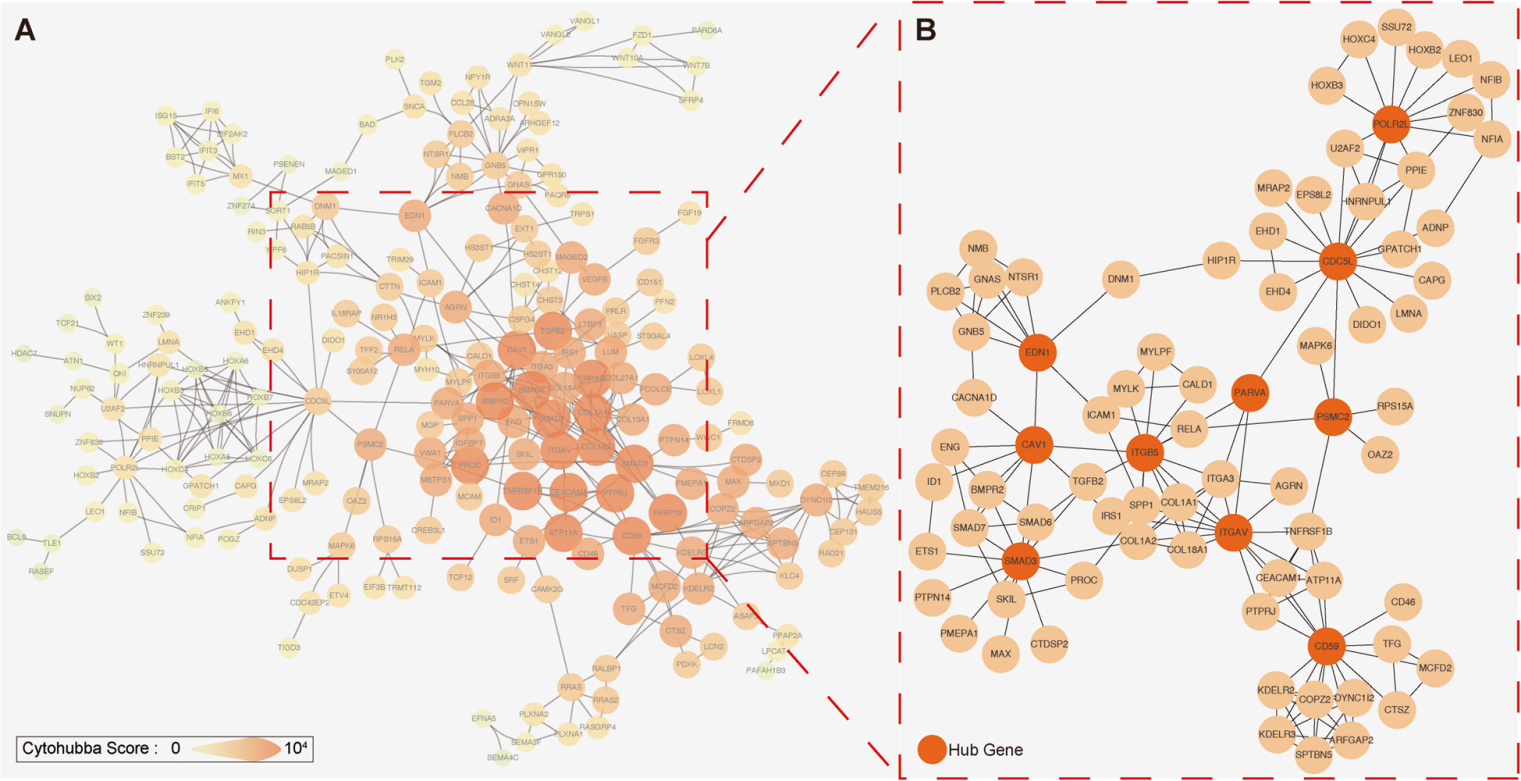
Protein–protein interaction network constructed with the differentially expressed genes. **a** PPI networks with confidence> 0.7. **b** PPI networks with hub genes

**Fig. 4:**
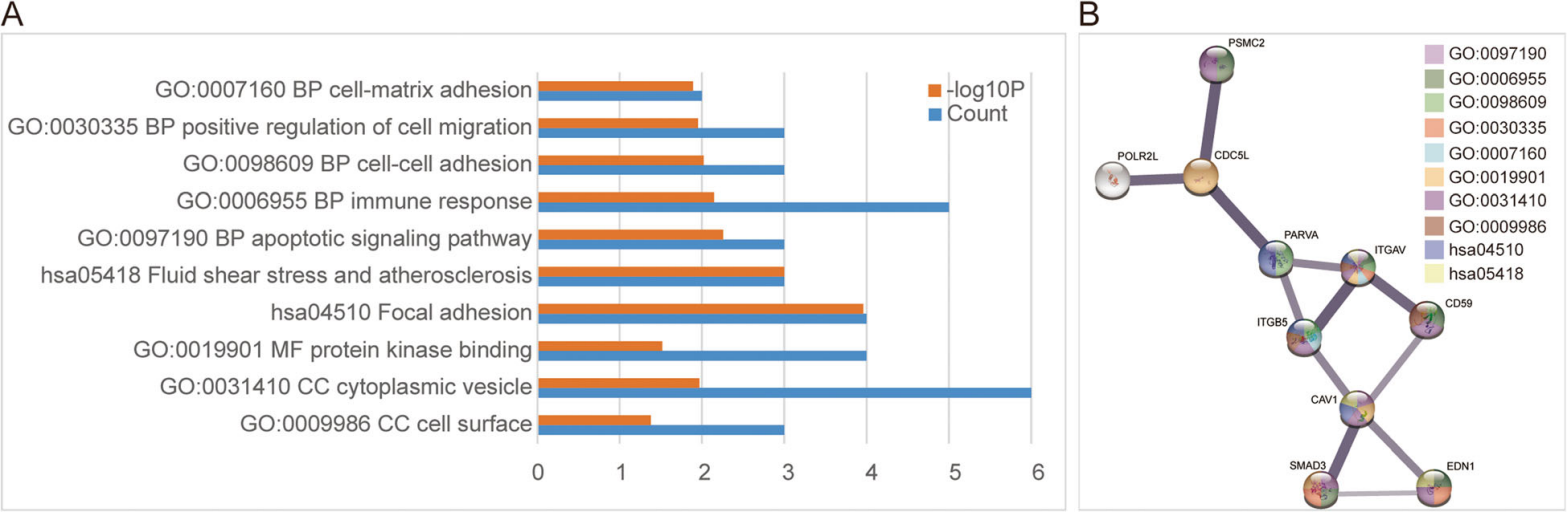
Hub genes enrichment results. **a** Significantly enriched GO terms and KEGG pathways of Hub genes. **b** Protein–protein interaction network of Hub genes

A protein interaction analysis was performed on the DEGs through STRING to obtain a PPI network, which contains 589 nodes and 440 edges. Highly connected nodes are critical to maintaining the stability of the entire protein network. Obtain the top 10 hub genes (Table 2) and their PPI network by Cytoscape algorithm (Fig. 3). These genes are Cell division cycle 5-like protein (*CDC5L*), Integrin alpha-V (*ITGAV*), CD59 glycoprotein (*CD59*), Endothelin-1 (*EDN1*), Caveolin-1 (*CAV1*), Alpha-parvin (*PARVA*), Integrin beta-5 (*ITGB5*), DNA-directed RNA polymerases I, II (*POLR2L*), Mothers against decapentaplegic homolog 3 (*SMAD3*), 26S proteasome regulatory subunit 7 (*PSMC2*). Hub genes were enriched and PPI again by STRING (Fig. 4). After enrichment, BP mainly focuses on cell adhesion and immune response, MF mainly includes protein kinase binding, CC includes cell surface and cytoplasmic vesicles, and pathway focuses on adhesion pathways and fluid shear forces.

**Table 2:** Top ten hub genes with higher score of connectivity

node_name	MCC	DMNC	MNC	Degree	EPC	BottleNeck	EcCentricity	Closeness	Radiality	Betweenness	Stress	ClusteringCoefficient	Total score
CDC5L	37	0.45	5	16	82.59	77	0.08	70.57	9.12	15,833	47,326	0.07	63,457.23
ITGAV	251	0.41	8	14	100.09	39	0.10	72.76	9.30	9450	37,160	0.26	47,105.29
CD59	847	0.71	6	15	97.09	32	0.10	64.82	8.82	8631	33,426	0.27	43,128.32
EDN1	29	0.45	5	8	85.45	48	0.09	63.64	8.94	8840	27,448	0.25	36,536.83
CAV1	17	0.47	4	9	94.12	37	0.10	65.29	9.05	6750	29,122	0.14	36,108.54
PARVA	7	0.46	3	4	90.13	13	0.09	64.87	9.17	6641	27,730	0.50	34,563.54
ITGB5	135	0.48	7	11	99.76	30	0.09	69.54	9.24	5305	23,774	0.29	29,441.69
POLR2L	33	0.45	5	12	74.03	18	0.07	59.69	8.49	6286	19,930	0.14	26,426.87
SMAD3	28	0.33	7	13	95.36	23	0.10	65.82	8.90	5886	19,698	0.12	25,825.54
PSMC2	6	0.00	1	6	75.37	98	0.09	64.25	9.09	7343	17,592	0.00	25,194.97

### Phenotypic identification of CTCs and prognostic value of hub gene

**Fig. 5:**
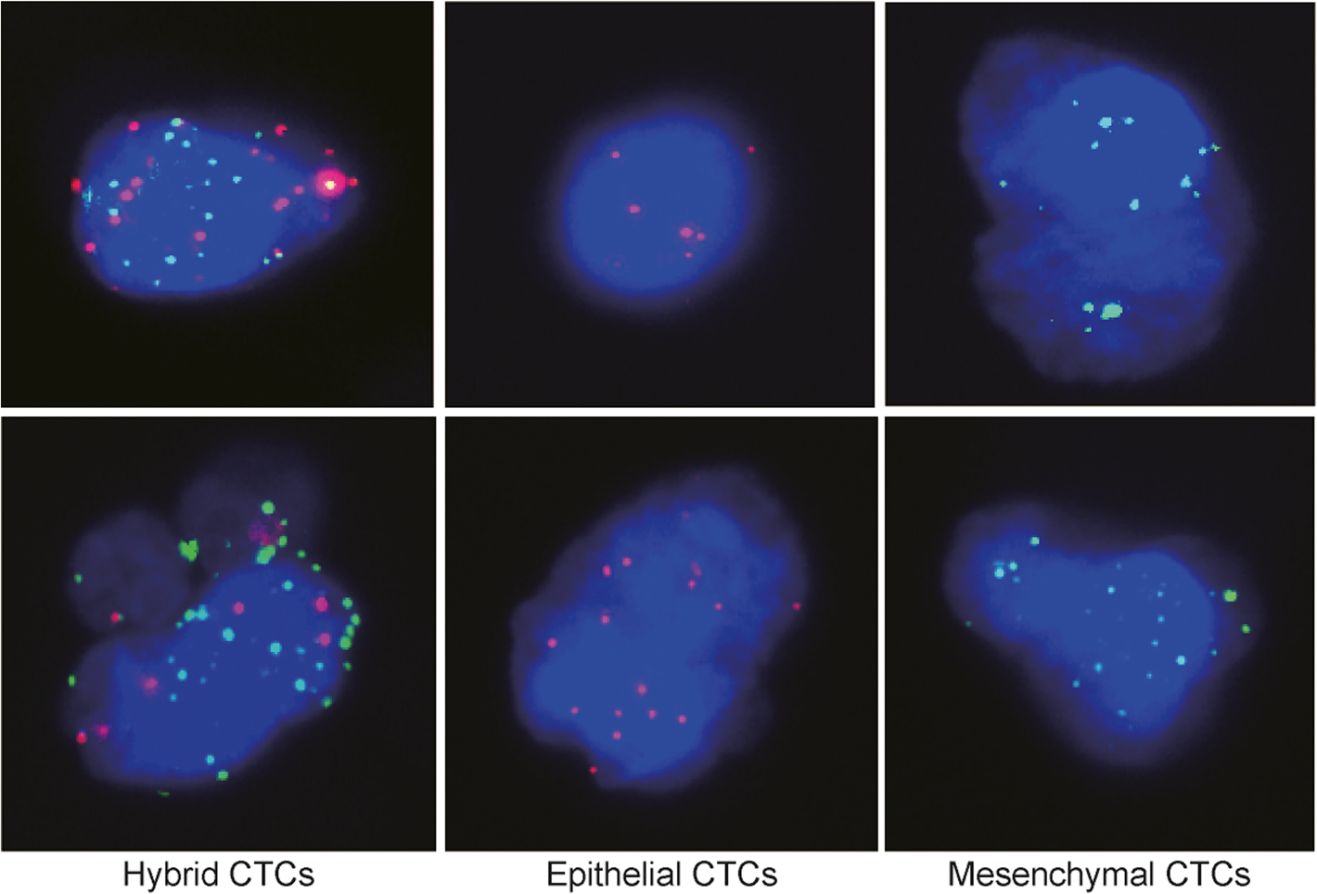
Different types of CTCs. CTCs were identified by the RNA in situ hybridization technique. Epithelial CTCs presented only Alexa Fluor 594 (Red color) labeled epithelial markers (EpCAM and CK8/18/19), mesenchymal CTCs exhibited only Alexa Fluor 488 (Green color) labeled mesenchymal markers (Vimentin and Twist), hybrid CTCs having both epithelial and mesenchymal markers were stained with both green and red immunofluorescent dyes

**Fig. 6:**
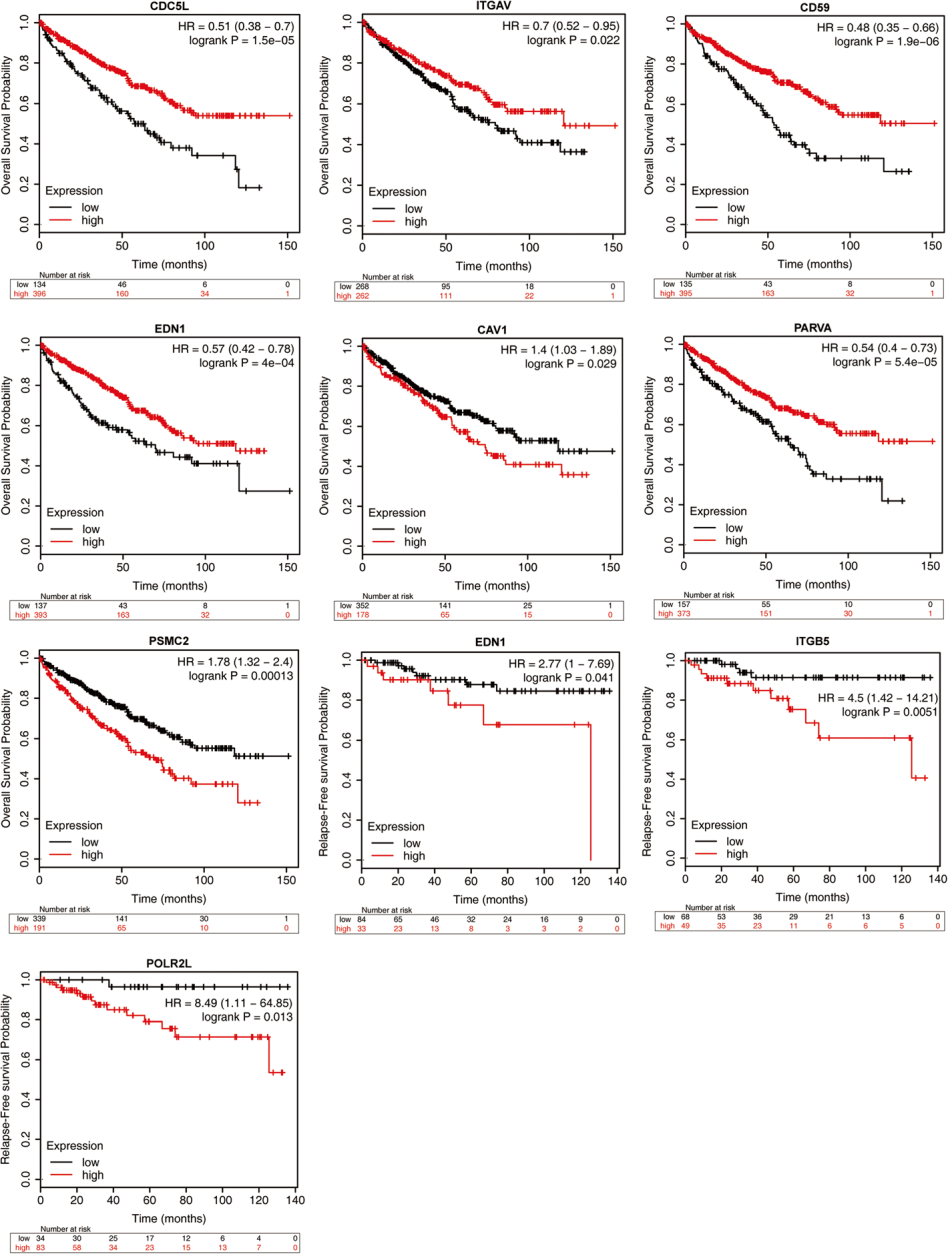
Kaplan–Meier overall survival analyses and relapse-free survival for the top ten hub genes expressed in renal clear cell carcinoma patients. *P* < 0.05 was considered statistically significant

Using the RNA in situ hybridization technique, CTCs obtained from the blood of kidney cancer patients can be divided into epithelial, interstitial, and mixed types (Fig. 5). At the same time, to determine the prognostic value of the top 10 hub genes, Kaplan–Meier plotter bioinformatics analysis was used to analyze overall survival and relapse-free survival of patients with renal clear cell carcinoma. Pan-cancer RNA-seq was used to measure expression of the top 10 hub genes in 530 kidney renal clear cell carcinoma samples. (Fig. 6). Among them, *CDC5L*, *ITGAV*, *CD59*, *EDN1*, *CAV1*, *PARVA*, and *PSMC2* have statistical significance in the prognosis of OS, and *EDN1*, *ITGB5*, *POLR2L* have statistical significance in the prognosis of RFS.

### Expression of PSMC2 in kidney cancer cell lines and tissues

**Fig. 7:**
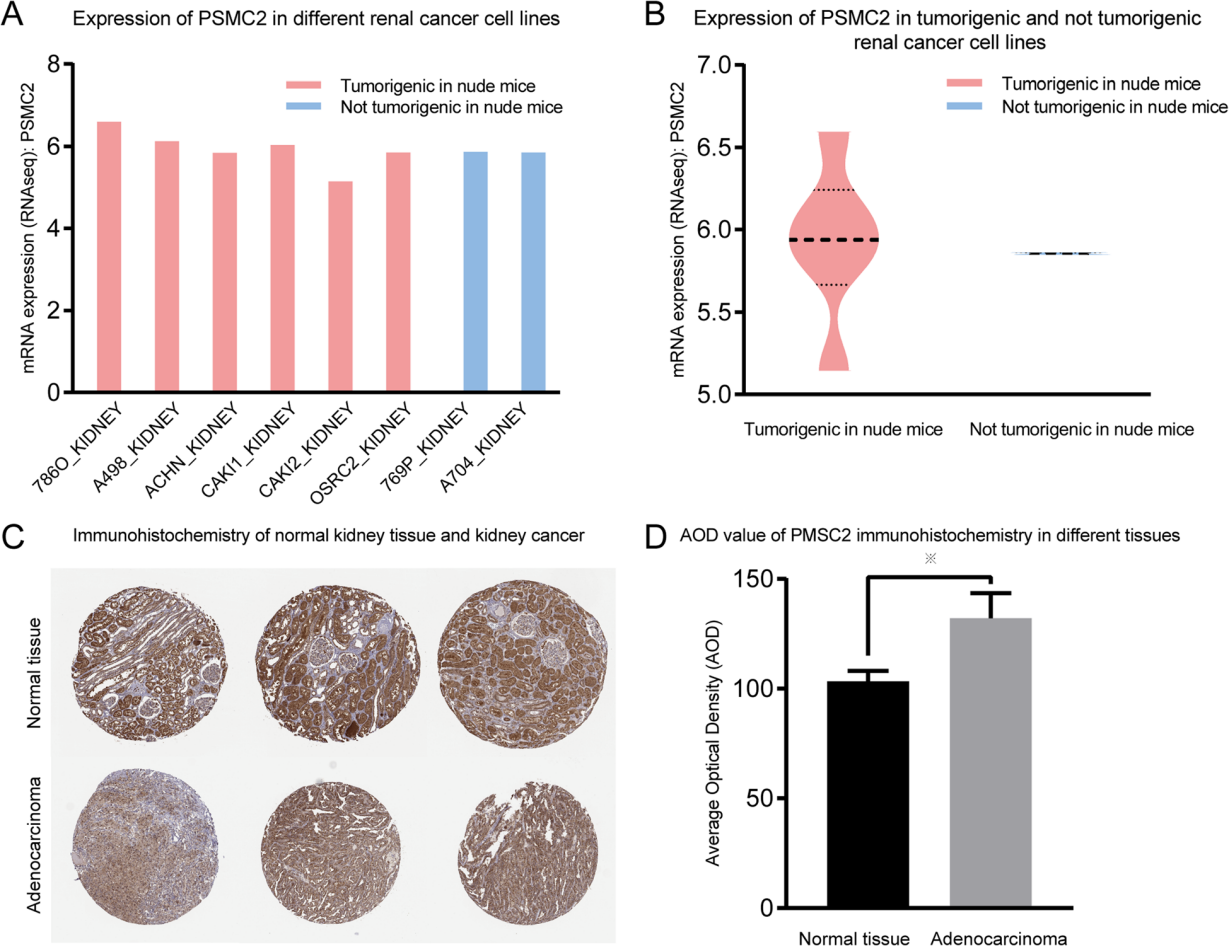
A Expression levels of PSMC2 in different renal cancer cell lines. B Comparison of the expression levels of PSMC2 between tumorigenic and non-tumorigenic renal cancer cell lines. C Immunohistochemistry of PSMC2 in normal kidney tissue and kidney cancer tissue. D Quantitative analysis of PSMC2 immunohistochemistry in normal kidney tissue and kidney cancer tissue(*P*<0.05)

A total of 1019 cell lines related to *PSMC2* RNAseq were obtained from the CCLE database, including 32 kidney cancer cell lines (Table S1). Klaudia conducted a systematic review of kidney cancer cell lines in 2016 [11], in the supplementary materials, the tumorigenicity of common kidney cancer cell lines in nude mice was summarized by Klaudia(Table S2). By combining *PSMC2* RNA expression data of common renal cancer cell lines and tumor formation data of nude mice, the correlation between *PSMC2* and invasiveness can be clarified, at the same time, the expression of *PSMC2* in kidney cancer tissue was higher than that in normal kidney tissue. (Fig. 7).

## Discussion

CTCs play a key role in the metastasis of cancer, with the development of new detection technologies in recent years, the enrichment and detection of CTCs have been improved, making CTCs a key object in tumor research [4, 12]. In this study, the genetic chip results of CTCs from different cancer sources were obtained through the GEO database, and gene expression and PPI networks were analyzed through bioinformatics tools. At the same time, clinical samples and prognostic results were verified, to identify potential genes and proteins closely related to CTCs.

In the process of CTCs leaving the primary focus and entering the blood circulation, there are mainly two ways: tumor cells leave the primary tumor and enter the blood circulation after epithelial-mesenchymal transition (EMT), and tumor cells enter the blood circulation through the gap between neovascular endothelial cells [13, 14]. EMT plays a key role in changes in cell polarity loss and decreased intercellular adhesion, leading the tumor cells to gain invasive [15]. As tumor cells develop EMT, the cell-to-cell bridge is lost, the expression of epithelial markers such as keratin, E-cadherin, and Epcam decreases, and the expression of mesenchymal marker vimentin increases, and its morphology changes, get rid of the primary tumor and obtain aggressive [16]. This is similar to our findings. Whether it is GO enrichment analysis of DEGs or hub genes (Fig. 2, Fig. 4), they all contain BP related to EMT. However, in Fig. 2, log10 (P) is partly used as the judgment result to be statistically significant, and the strength of the argument is poor. The original GO enrichment analysis table (Table S4) generated by metascape tool contains the log10(q) related information of GO enrichment. “Log10(P)” is the *p*-value in log base 10. “Log10(q)” is the multi-test adjusted p-value in log base 10. A total of 234 enriched results of Log10(q) are statistically significant, and a large number of them contain GO results related to epithelial-mesenchymal transition. Such as epithelial differentiation, mesenchymal differentiation, cytoskeleton, cell adhesion, etc., and also includes biological processes related to downstream effects of EMT such as cell migration, invasion, etc. We also confirmed from clinical samples that CTCs do have EMT-related changes (Fig. 5).

When CTCs enter the blood circulation, they are affected by blood flow shear force, anoikis, and immune cell recognition and killing [17], which causes CTCs to have a shorter half-life in the blood, about 1–2 h [18]. The shear force will cause a large number of death of circulating tumor cells. Some studies have shown that mesenchymal tumor cells undergoing EMT are more resistant to shear force than epithelial circulating tumor cells [19]. CTCs inhibit caspase-related apoptosis by activating tropomyosin-associated kinase B (TrkB), thereby combating anoikis [20]. The selective expression of CD47 and other proteins by CTCs can reduce the killing of CTCs by immune cells and improve the viability of CTCs in the bloodstream [21]. This is also in line with our findings. In our enrichment of DEGs and hub genes, included signal pathways of fluid shear forces, molecular functions of protein kinases, immune responses, and biological processes of apoptosis (Fig. 2, Fig. 4).

Proteasome 26S subunit ATPase 2 (*PSMC2*) is a basic member encoding the 19S proteasome and plays a key role in the assembly of the 19 s and 26 s proteasomes, further regulating the protein degradation of eukaryotic cells [22–24]. Studies have shown that in ovarian cancer cells, inhibiting *PSMC2* expression can inhibit cell proliferation [25]. In osteosarcoma samples, *PSMC2* was highly expressed, and after silencing *PSMC2*, osteosarcoma cell lines (SaoS-2 and MG-63) showed cell proliferation inhibition, cell cycle arrest, and increased apoptosis, these changes were further confirmed in nude mice [26]. Similar findings have been confirmed in colorectal cancer cells, pancreatic cancer, and acute promyelocytic leukemia cells [27–29]. Further research suggests that miR-630 can act on *PSMC2* and regulate cell growth, colony formation, migration and invasion, and EMT processes, at the same time, high *PSMC2* expression is associated with poor prognosis [30]. Besides, proteasome inhibitor bortezomib is used as a first-line treatment in multiple myeloma [31]. This is similar to our findings. In renal cell carcinoma patients, *PSMC2* is a poor prognostic factor for OS (HR = 1.78, 95% CI = 1.32–2.4, *P* < 0.001). At the same time, the average RNA expression of *PSMC2* in renal cancer cells with tumorigenicity was higher than that of non-tumorigenic kidney cancer cells, but the data were too few to judge statistical significance. At the tissue level, the expression of *PSMC2* in kidney cancer tissue was significantly higher than that in normal kidney tissue. Indirect evidence at the cellular and tissue level suggests to a certain extent that *PSMC2* plays a role in promoting tumors in renal cell carcinoma. Therefore, *PSMC2* is expected to become a prognostic factor and therapeutic target for renal cancer.

Although we obtain different genes by combining different data sets, and the existing research has adopted the similar merging method [32]. However, it is undeniable that such a treatment method needs to consider the impact of batch effects on the results. Batch effects, which occur because measurements are affected by laboratory conditions, reagent lots and personnel differences [33]. Combining or merging data from different microarray gene expression experiments for integrative analysis suffers from batch effects and it still is a challenging and difficult problem to be solved in computational biology [34]. In high-throughput data, if not handled properly, batch effects may lead to erroneous biological conclusions [35]. When we analyze the data, we normalized the data. Including a normalization step is now standard in data analysis of gene expression experiments [36]. However, normalization does not remove batch effects, which affect specific subsets of genes and may affect different genes in different ways [33]. Therefore, in the subsequent analysis, we introduced the R limma package, Law mentioned this differential expression methods for RNA-seq experiments of arbitrary complexity, for example experiments with multiple treatment factors, batch effects or numerical covariates [37]. However, since our experiment cannot change the high-throughput experimental design that has been included in the data set, which is high-throughput experiments should be designed to distribute batches and other potential sources of experimental variation across biological group [38]. Therefore, this limma package cannot effectively remove the batch effect of this research. Real biological effects genes and technical artifacts genes may exist in the DEG of each cancer type, and technical artifacts account for the majority. It is inevitable, Jeffrey T. Leek performs batch analysis on 9 high-throughput data (such as microarray, sequencing and mass spectrometry) from public databases. It was discovered that batch effects for all of these data sets, and substantial percentages (32.1–99.5%) of measured features showed statistically significant associations with processing date, irrespective of biological phenotype, this suggests that technical variability was more influential than biological variability across a range of experimental conditions and technologies [33]. Similarly, Margus Lukk constructed a global gene expression map by integrating microarray data from 5372 human samples representing 369 different cell and tissue types, disease states and cell lines. However, the authors did not explicitly address the batch effect problem, which turns to be a critical obstacle for genomic data integration [39]. Therefore, in the subsequent analysis, we adopted methods such as taking the intersect between the three lists and running network analysis to define the top 10 most important to reduce the possible impact of the batch effect. The key gene is then verified at the tissue level and clinical samples.

In our study, it was clarified that the functional enrichment of DEGs between CTCs and primary tumors focused on the acquisition of invasiveness and the biological processes of survival in the blood. At the same time, the clinical data were used to corroborate the results of the bioinformatics analysis, and the possibility of *PSMC2* as a prognostic factor and treatment target was discussed. However, this study has the disadvantages of a small number of included specimens and insufficient types of tumors. In subsequent experiments, on the one hand, data from multiple centers and multiple cancer species can be collected to overcome the shortcomings of this experiment. On the other hand, the obtained hub gene can be verified by cell and molecule experiments, thereby laying a foundation for clarifying key molecular targets of CTCs.

## Conclusion

Based on the bioinformatics analysis of three cancers in the GEO database, we obtained 589 common genes with different expressions between the CTCs and the primary tumor. Among these genes, GO / KEGG enrichment analysis suggests that the DEGs are mainly concentrated in CTCs that gain invasiveness through EMT, and fight against shear forces, apoptosis and immune responses in the blood circulation, and are verified by clinical samples from renal cancer patients. We also analyzed the *PSMC2* in the hub gene and found that overexpression of *PSMC2* in various cancers is associated with poor prognosis, clarified its value as a prognostic factor and therapeutic target for renal cancer.

## Supplementary information

**Additional file 1: Table S1.**

**Additional file 2: Table S2.**

**Additional file 3: Table S3.**

**Additional file 4: Table S4.**

## Data Availability

The datasets analyzed during the current study are available in the GEO database through GEO accession numbers GSE82198, GSE99394, GSE31023, GSE65505, GSE67982, GSE76250, GSE50746.

## Abbreviations

CTCs: Circulating tumor cells
GEO: Gene Expression Omnibus
DEGs: Differentially expressed genes
GO: Gene Ontology
KEGG: Kyoto Encyclopedia of Genes and Genomes
PPI: Protein-protein interaction
EMT: Epithelial-mesenchymal transition
BP: Biological processes
CC: Cell components
MF: Molecular functions
PSMC2: Proteasome 26S subunit ATPase 2
