# Supplementary material for: Identification of key genes and functions of circulating tumor cells in multiple cancers through bioinformatic analysis
Source: BMC Med Genomics. 2020 Sep 24;13:140. doi: 10.1186/s12920-020-00795-w (PMC7513313; doi:10.1186/s12920-020-00795-w)
Supplement: Supplementary file 1 — Additional file 1: Table S1. [file 12920_2020_795_MOESM1_ESM.docx]

| Gene | PSMC2 |
| --- | --- |
| 769P_KIDNEY | 5.864183594 |
| 786O_KIDNEY | 6.596077806 |
| A498_KIDNEY | 6.124110897 |
| A704_KIDNEY | 5.848631058 |
| ACHN_KIDNEY | 5.839730677 |
| BFTC909_KIDNEY | 4.830143016 |
| CAKI1_KIDNEY | 6.033344691 |
| CAKI2_KIDNEY | 5.143789254 |
| CAL54_KIDNEY | 5.614607634 |
| HEKTE_KIDNEY | 5.646990517 |
| KMRC1_KIDNEY | 6.474158587 |
| KMRC2_KIDNEY | 5.874787313 |
| KMRC20_KIDNEY | 6.486509887 |
| KMRC3_KIDNEY | 5.104888882 |
| OSRC2_KIDNEY | 5.84441921 |
| RCC10RGB_KIDNEY | 4.848435166 |
| SKRC20_KIDNEY | 6.251040695 |
| SLR20_KIDNEY | 5.101260958 |
| SLR21_KIDNEY | 5.361773274 |
| SLR23_KIDNEY | 6.187772502 |
| SLR24_KIDNEY | 5.528414047 |
| SLR25_KIDNEY | 6.059484253 |
| SLR26_KIDNEY | 6.105377683 |
| SNU1272_KIDNEY | 5.240981402 |
| SNU349_KIDNEY | 5.62906274 |
| TUHR10TKB_KIDNEY | 6.862774588 |
| TUHR14TKB_KIDNEY | 5.729470555 |
| TUHR4TKB_KIDNEY | 6.208773158 |
| UO31_KIDNEY | 5.845165933 |
| UOK101_KIDNEY | 5.788838688 |
| VMRCRCW_KIDNEY | 5.680558694 |
| VMRCRCZ_KIDNEY | 5.605430573 |
