# Supplementary material for: Identification of key genes and functions of circulating tumor cells in multiple cancers through bioinformatic analysis
Source: BMC Med Genomics. 2020 Sep 24;13:140. doi: 10.1186/s12920-020-00795-w (PMC7513313; doi:10.1186/s12920-020-00795-w)
Supplement: Supplementary file 2 [file 12920_2020_795_MOESM3_ESM.docx]

Dataset ID	Cancer	GEO accession	metastasis	Gender	Primary tumor subtype	age (yrs)	menopause status (0=pre, 1=post)	size (cm)	tumor grade	positive lymph nodes	ki67(%)
GSE99394	Breast cancer	GSM2643403	Negative	female	TNBC	/	/	/	/	/	/
		GSM2643404	Negative	female	ER-/PR-/HER2+	/	/	/	/	/	/
		GSM2643405	Negative	female	TNBC	/	/	/	/	/	/
		GSM2643406	Negative	female	ER+/PR-/HER2-	/	/	/	/	/	/
		GSM2643407	Negative	female	TNBC	/	/	/	/	/	/
		GSM2643408	Positive	female	ER+/PR+/HER2-	/	/	/	/	/	/
		GSM2643409	Positive	female	ER+/PR-/HER2-	/	/	/	/	/	/
		GSM2643410	Positive	female	ER+/PR-/HER2-	/	/	/	/	/	/
		GSM2643411	Positive	female	ER+/PR+/HER2-	/	/	/	/	/	/
		GSM2643412	Positive	female	TNBC	/	/	/	/	/	/
GSE65505	Breast cancer	GSM1598917	Negative	female	ER+/PR+/HER2—	/	/	/	/	/	/
		GSM1598918	Negative	female	ER+/PR+/HER3—	/	/	/	/	/	/
		GSM1598919	Negative	female	ER+/PR+/HER4—	/	/	/	/	/	/
GSE67982	Breast cancer	GSM1660248	/	female	ER+/PR+/HER2+	/	/	/	/	/	/
		GSM1660249	/	female	ER+/PR+/HER3+	/	/	/	/	/	/
		GSM1660250	/	female	ER+/PR+/HER4+	/	/	/	/	/	/
GSE76250	Breast cancer	GSM1977647	Positive	female	TNBC	41	0	4	3	1	15
		GSM1977648	Negative	female	TNBC	59	1	1.5	2	0	95
		GSM1660248	Positive	female	TNBC	83	1	3	0	12	95
GSE82198	Colon cancer	GSM2186367	/	/	/	/	/	/	/	/	/
		GSM2186368	/	/	/	/	/	/	/	/	/
		GSM2186369	/	/	/	/	/	/	/	/	/
		GSM2186370	/	/	/	/	/	/	/	/	/
		GSM2186371	/	/	/	/	/	/	/	/	/
		GSM2186372	/	/	/	/	/	/	/	/	/
GSE31023	Colorectal cancer	GSM768757	/	/	/	/	/	/	/	/	/
		GSM768758	/	/	/	/	/	/	/	/	/
		GSM768759	/	/	/	/	/	/	/	/	/
		GSM768760	/	/	/	/	/	/	/	/	/
		GSM768761	/	/	/	/	/	/	/	/	/
		GSM768762	/	/	/	/	/	/	/	/	/
GSE50746	Colorectal cancer	GSM1227930	/	male	/	80	/	/	/	/	/
		GSM1227932	/	female	/	79	/	/	/	/	/
		GSM1227934	/	male	/	83	/	/	/	/	/
		GSM1227936	/	male	/	85	/	/	/	/	/
		GSM1227938	/	male	/	85	/	/	/	/	/
		GSM1227942	/	female	/	75	/	/	/	/	/
		GSM1227946	/	female	/	80	/	/	/	/	/

"/" : Not mentioned
